# Successful Phenotype Improvement following Gene Therapy for Severe Hemophilia A in Privately Owned Dogs

**DOI:** 10.1371/journal.pone.0151800

**Published:** 2016-03-24

**Authors:** Mary Beth Callan, Mark E. Haskins, Ping Wang, Shangzhen Zhou, Katherine A. High, Valder R. Arruda

**Affiliations:** 1 Department of Clinical Studies, School of Veterinary Medicine, University of Pennsylvania, Philadelphia, Pennsylvania, United States of America; 2 Department of Pathobiology, School of Veterinary Medicine, University of Pennsylvania, Philadelphia, Pennsylvania, United States of America; 3 Center for Cellular and Molecular Therapeutics, The Children’s Hospital of Philadelphia, Philadelphia, Pennsylvania, United States of America; 4 Department of Pediatrics, Perelman School of Medicine, University of Pennsylvania, Philadelphia, Pennsylvania, United States of America; Justus-Liebig-University Giessen, GERMANY

## Abstract

Severe hemophilia A (HA) is an inherited bleeding disorder characterized by <1% of residual factor VIII (FVIII) clotting activity. The disease affects several mammals including dogs, and, like humans, is associated with high morbidity and mortality. In gene therapy using adeno-associated viral (AAV) vectors, the canine model has been one of the best predictors of the therapeutic dose tested in clinical trials for hemophilia B (factor IX deficiency) and other genetic diseases, such as congenital blindness. Here we report our experience with liver gene therapy with AAV-FVIII in two outbred, privately owned dogs with severe HA that resulted in sustained expression of 1–2% of normal FVIII levels and prevented 90% of expected bleeding episodes. A Thr62Met mutation in the *F8* gene was identified in one dog. These data recapitulate the improvement of the disease phenotype in research animals, and in humans, with AAV liver gene therapy for hemophilia B. Our experience is a novel example of the benefits of a relevant preclinical canine model to facilitate both translational studies in humans and improved welfare of privately owned dogs.

## Introduction

Hemophilia is an X-linked bleeding disorder characterized by deficiency in the clotting activity of factor VIII (hemophilia A, HA) or factor IX (hemophilia B, HB), key components of the coagulation cascade. These coagulopathies are clinically indistinguishable. However, HA represents 80% of all human hemophilia cases, occurring in ~1 in 10,000 live male births worldwide, and in 30% of the cases there is no family history of HA. Patients with severe HA have residual FVIII activity <1% of normal, resulting in recurrent spontaneous bleeding episodes from early in life, mostly into joints (hemarthrosis) and soft tissues, but also into closed spaces, such as the brain and retroperitoneum, leading to increased morbidity and mortality. Notably, residual FVIII levels of 1–5% (moderate disease) result in significant phenotype improvement by decreasing spontaneous bleeds, and those with mild disease (FVIII 6–30%) experience bleeding mostly secondary to trauma or surgical intervention [1]. Current treatment of HA is based on plasma-derived or recombinant protein replacement therapy to control bleeding or prevent bleeding prior to invasive procedures. Over the last decades it became clear that prophylactic therapy with protein injection (3 times or more/week) is beneficial in minimizing the bleeding phenotype and preventing the joint disease. This therapeutic strategy is often recommended for boys around 2 years of age or after the first episode of bleeding [2,3] and more recently, throughout adult life [4]. Limitations of protein-based therapy include the short half-life of FVIII (8–12 hours) necessitating frequent intravenous injections and the need for intravascular catheter placement in the pediatric population, which is unfortunately complicated by infection, bleeding, and thrombosis [5,6]. In addition, one of the major complications of protein replacement therapy is the development of inhibitory alloantibodies to the replacement protein that affect 20–30% of severe HA patients [6,7] that neutralize the pro-hemostatic effect of FVIII; inhibitors are also present in 9% of non-severe HA [8]. Despite the efficacy of replacement therapy for hemophilia, it is estimated that only 20% of patients worldwide have access to the treatment, mostly due to the high cost [9]. Therefore, the development of alternative therapeutic strategies is needed for hemophilia. Gene therapy has gained growing interest since early data showed efficient expression of FIX reaching levels of 12% of normal following delivery of an adeno-associated viral (AAV) vector encoding the human F9 gene for hepatocyte-restricted expression [10]. These data were based on the use of a natural canine model of severe HB that accurately predicted the therapeutic dose of vector required in humans [11]. Moreover, recent data showed long-term expression of FIX in severe HB patients following a single injection of AAV-FIX at levels of 1–6% in a dose-dependent manner, and in 4 of 7 subjects prophylactic therapy was discontinued [12]. Together, these data further motivate the development of translational studies of AAV-based therapy for liver expression of FVIII for HA. Fortunately, the availability of dogs with severe HA that mimics the disease phenotype and the immune responses to canine FVIII protein provides an excellent preclinical model [13,14].

The rationale for the initial focus on the development of AAV-FIX to treat HB, the least common form of hemophilia, was due to the small size of the FIX cDNA being within the packaging capacity of the AAV vector (4.7 Kb). Secondly, the risk of development of antibodies to FIX is remarkably lower than to FVIII (3% versus 20%), and it is reasonable to expect that similar complications should be anticipated in gene-based strategies.

Continuous efforts by many groups of investigators are now supporting translational studies using AAV-FVIII [15–18]. Factor VIII is a large protein (280 kDa) compared to FIX (55 kDa). Two forms of recombinant FVIII are used clinically and are similarly efficacious and safe. One is the full-length FVIII and the other is a short version lacking most of the B-domain, which comprises 40% of the protein and is not required for the coagulation activity; the latter is named B-domain deleted (BDD) form of FVIII.

We have used canine HA models (n = 13) from two distinct colonies for AAV liver gene therapy encoding canine FVIII-BDD (cFVIII-BDD) [14,16,19]. Long-term follow-up showed vector dose-dependent expression of therapeutic levels of cFVIII-BDD ranging from >1%-10%, with follow-up of 1 to 5 years (cumulative 25 years/9 dogs, ongoing observations). The disease phenotype was clearly improved by a greater than 90% reduction in bleeding episodes expected for untreated dogs. We also developed a system for the expression of recombinant cFVIII-BDD protein, and extensive characterization at the biochemical level showed proper processing with enhanced biologic activity compared to humans [20]. Infusion of the cFVIII-BDD protein in neonate and adult HA dogs showed that the canine protein corrected the hemophilia phenotype and exhibited a half-life comparable to the human protein, without increased immunogenicity upon repeated injections. Collectively, these data formed the basis of our proposal to extend the benefits of liver gene therapy to privately owned dogs with severe HA. Currently, these dogs are treated with plasma-derived products to control bleeds, which is challenging and associated with substantial morbidity. Here we report the long-term outcomes of two dogs administered intravenous AAV-cFVIII-BDD and maintained in their home environment.

## Materials and Methods

### Privately owned dogs

This study was approved by Institutional Animal Care and Use Committee from Matthew J. Ryan Veterinary Hospital of the University of Pennsylvania and The Children’s Hospital of Philadelphia. After obtaining signed owner informed consent, two privately owned young adult male dogs with severe HA were identified for investigational treatment based on their frequent bleeding episodes requiring transfusion support (Table 1). Dog 1, a German shepherd mixed breed, had 2 male littermates that were euthanized at 7 weeks of age due to severe bleeding; a diagnosis of hemophilia A was confirmed in one puppy based on plasma FVIII coagulant activity <1%. Dog 2, a Staffordshire bull terrier, was the result of a mating of two unrelated dogs within the household; there was no history of bleeding among his only sibling (female), dam, and sire or their families.

**Table 1:** Bleeding episodes and transfusion events prior to gene therapy in privately owned dogs with severe hemophilia A.

Age	Bleeding event	Spontaneous or traumatic	Transfusion support
**Dog 1**			
8 weeks	Hematoma in hindlimb	Spontaneous	None
9 weeks	Suspected hemothorax (cough and dyspnea)	Spontaneous	None
12 weeks	Hematoma on head	Spontaneous	CRYO^a^
20 weeks	Oral bleeding and ecchymoses on abdomen	Spontaneous	CRYO
21 weeks	Oral bleeding following loss of tooth	Spontaneous	CRYO
23 weeks	Hematomas on leg and neck	Spontaneous	CRYO
25 weeks	Hemarthrosis and hematoma on thorax; blood loss anemia	Spontaneous	CRYO
32 weeks	Scrotal hemorrhage after castration	Traumatic	CRYO, PRBCs^b^
40 weeks	Hemarthrosis and hematoma over shoulder	Spontaneous	CRYO
41 weeks	Hematoma on neck; blood loss anemia	Spontaneous	CRYO, PRBCs
46 weeks	Recurring hematoma over shoulder	Spontaneous	CRYO
50 weeks	Recurring hematoma over shoulder	Spontaneous	CRYO
**Dog 2**			
8 weeks	Hematoma on head; blood loss anemia	Spontaneous	FWB^c^
10 weeks	Hematoma in hindlimb	Spontaneous	FFP^d^
23 weeks	Hematoma in hindlimb and epistaxis; blood loss anemia	Spontaneous	FWB
11 months	Severe melena following corticosteroid administration for allergies	Spontaneous	FWB
11.5 months	Hematoma in forelimb	Spontaneous	FWB
12–14 months	Recurring hematoma in hindlimb	Spontaneous	FWB or FFP on 5 separate occasions
17 months	Bleeding from sutures placed for conjunctival flap (corneal ulcer)	Traumatic	FFP
19 months	Hemarthrosis	Spontaneous	FFP
20 months	Epistaxis and gingival bleeding	Spontaneous	None
21 months	Epistaxis and gingival bleeding	Spontaneous	None
22 months	Hematoma in hindlimb	Spontaneous	FFP on 5 separate occasions during the month
24 months	Bleeding after microchip placement	Traumatic	None
25 months	Hematoma in hindlimb	Spontaneous	None

^a^CRYO, cryoprecipitate

^b^PRBCs, packed red blood cells

^c^FWB, fresh whole blood

^d^FFP, fresh frozen plasma.

Results of initial screening coagulation testing performed by outside reference laboratories for both dogs included a normal prothrombin time, prolonged activated partial thromboplastin time, increased plasma von Willebrand factor antigen concentration (Dog 1, 248%; Dog 2, 259%; reference interval 70–180%), and normal FIX activity (Dog 1, 115%; Dog 2, 52%; reference interval 50–150%).

### Canine FVIII expression cassette

To overcome the limited packaging capacity of AAV vectors (4.7 Kb) for the expression of the cFVIII-BDD (4.5 Kb) alone without promoter, intron sequences, and regulatory elements, an early approach consisted of two separate vectors for the expression of the light chain (LC) and the heavy chain (HC), with both transgenes under the control of a hepatocyte-specific promoter, thyroxine-binding globulin gene promoter/enhancer [16].

### Recombinant AAV vector production

Recombinant AAV vectors were produced by a triple transfection protocol as previously described, using plasmids expressing cFVIII-BDD in separate vectors, a second plasmid supplying adenovirus helper functions, and a third plasmid containing the AAV2 *rep* gene and the AAV8 or *cap* genes [14]. Vectors were purified by repeated cesium chloride density gradient centrifugation. Vector titers were obtained by Taqman PCR (Applied Biosystems, Foster City, CA).

### Detection of pre-existing neutralizing antibodies to AAV8 serotype

To detect neutralizing antibody responses against AAV8 capsid proteins we used serial dilution of serum samples collected before and after vector delivery, as previously described [10] with modifications for AAV8 [21] and for canine sera. Data are reported as the serum dilution.

### Canine FVIII antigen, activity, and antibody assays

Canine FVIII clotting activity was determined by Chromogenix Coatest SP4 FVIII (Diapharma, Lexington, MA) and cFVIII antigen levels were analyzed by ELISA as previously described [14]. Anti-cFVIII-BDD antibodies were detected by a mixing test or Bethesda assay and by cFVIII-BDD specific IgG antibodies using anti- cFVIII-BDD ELISA as previously described [14].

### Animal procedures

The two young adult male HA dogs were administered 2.5 x 10^13^ vg/kg of AAV8-cFVIII-LC and 2.5 x 10^13^ vg/kg AAV8-cFVIII-HC via the saphenous vein in a total volume of 10 mL/kg body weight PBS. We expressed, purified, and characterized the recombinant cFVIII-BDD protein, which was tested in research dogs with HA and had an excellent efficacy and safety profile [14]. This protein was administered at doses of 50 IU/kg immediately prior to the vector injection, as well as following 2 subsequent bleeding episodes (Dog 1). Dogs were hospitalized for 2 days after treatment for monitoring.

### Adverse events

Complete physical examinations were performed prior to vector administration and at several time points during the two days of hospitalization. Hematologic and comprehensive biochemical analyses of blood and serum samples were performed pre-treatment and at several time points weeks to months following vector administration.

### Molecular diagnosis of hemophilia A

DNA was extracted from peripheral blood samples from both dogs and from the dam, sire, and 2 female siblings of Dog 1, as well as a clinically normal dog. Twenty two pairs of oligonucleotide primers (sequences and PCR conditions available upon request) were used to amplify the coding regions and exon-intron boundaries of the cFVIII gene, comprised of 26 exons, for the dogs with HA and a clinically normal dog. Once a mutation in exon 2 was identified for Dog 1, exon 2 was amplified by PCR for this dog’s family members.

To further determine the impact of the amino acid substitution on the structure and function of the F8 protein (NCBI protein ID NP_001003212.1, http://www.ncbi.nlm.nih.gov), the F8 amino acid sequence around the substitution site of Dog 1 was aligned and compared with that of normal dogs and other mammalian species. Also, an amino acid destructive analysis was performed on the F8 protein using the sorting intolerant from tolerant (SIFT) algorithm and protein variation effect analyzer (PROVEAN) software (The J. Craig Venter Institute, Rockville, MD, http://sift.jcvi.org). The analysis scale ranges from 0.00 (unacceptable, this amino acid substitution renders the protein completely nonfunctional) to 1.00 (normal, the substitution has no effect); the threshold for intolerance is ≤0.05, where an amino acid score ≤0.05 is predicted to be deleterious.

## Results

### Pre-treatment screening

**Fig 1:**
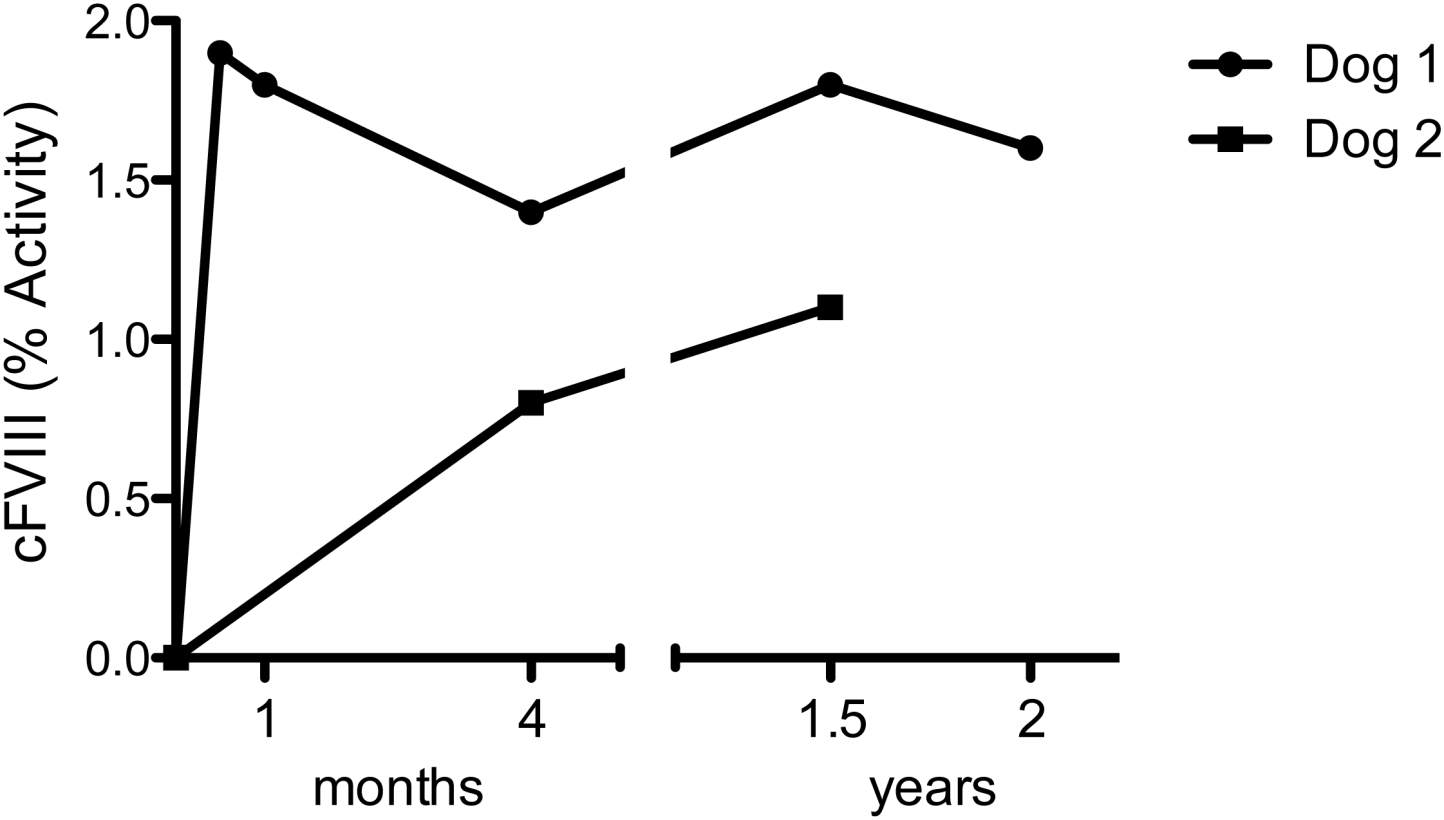
Time course of canine FVIII activity following intravenous injection of AAV8-cFVIII-BDD in severe hemophilia A dogs.

**Fig 2:**
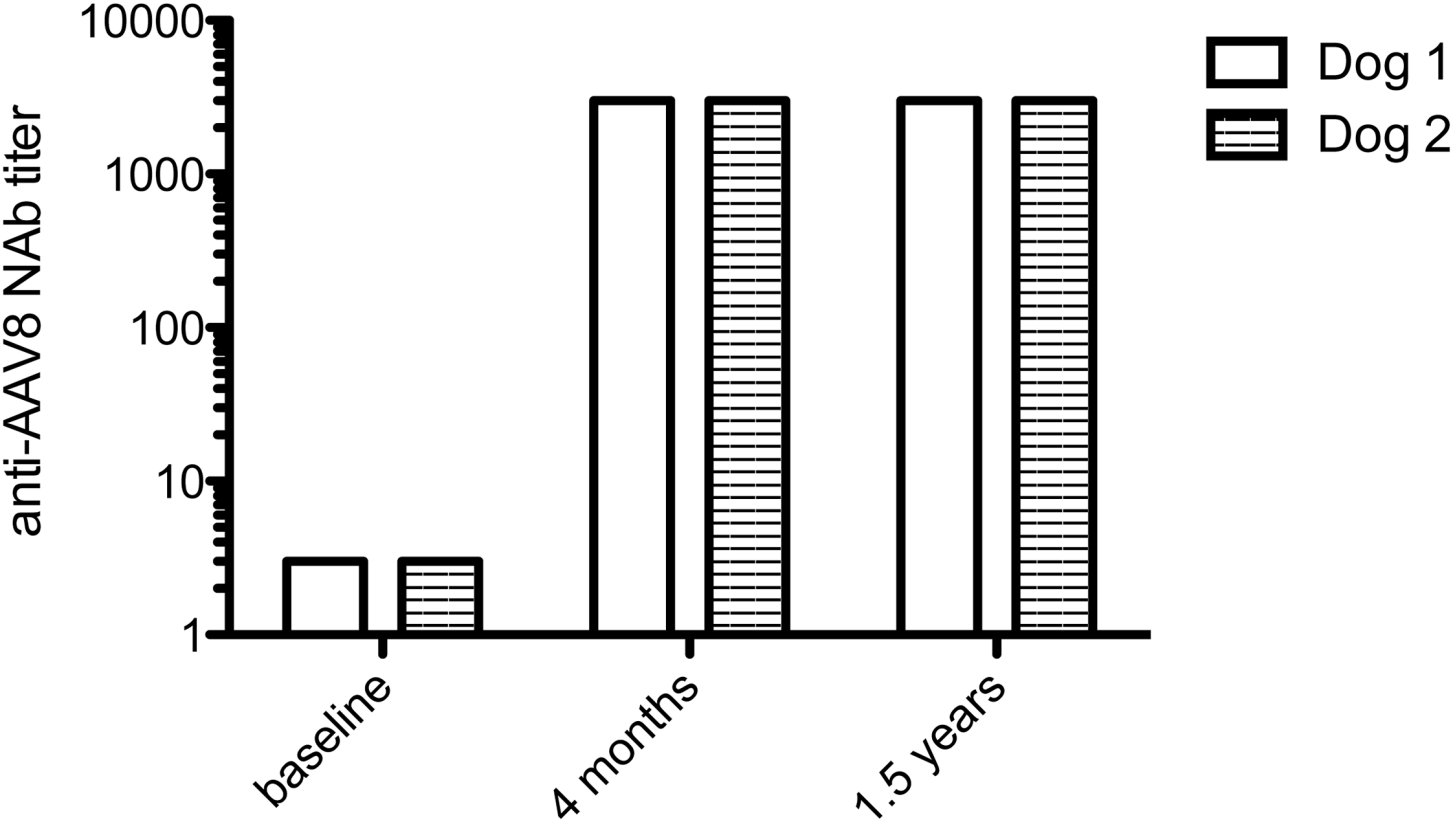
Humoral response to AAV8 capsid in hemophilia A dogs before and after intravascular delivery of AAV8-cFVIII-BDD.

The bleeding history and transfusion events prior to gene therapy of both dogs are shown in Table 1 and Fig 1. Baseline plasma FVIII coagulant activity and antigen were determined to be <1% in both dogs. Because of the previous exposure to multiple plasma products, the dogs were also screened for the presence of neutralizing and non-neutralizing antibodies to cFVIII prior to AAV injection, and both tested negative. Neither dog had detectable circulating cFVIII-BDD by activity or antigen assay in blood samples collected at baseline. There were very low levels of detectable neutralizing antibodies to the AAV8 capsid (titer range: 1:1–1:3) (Fig 2). Thus, both dogs were eligible for AAV8-cFVIII-BDD liver gene therapy since only titers above 1:5 prevent liver gene transfer [12,22,23].

### Molecular characterization of hemophilia A

Comparison of DNA sequences of the entire coding region and exon-intron boundaries of the *F8* gene from the 2 affected dogs to the published canine genome sequence revealed a single nucleotide substitution in exon 2, 185C>T, resulting in a substitution of threonine 62 by methionine (Thr62Met) in Dog 1. Sequences from the clinically normal sire of Dog 1 and an unrelated normal dog were wild-type, while the dam and two female siblings were heterozygous for the mutation. No mutation was identified in the *F8* gene from Dog 2, including the inversion of intron 22. Multiple alignments of the F8 protein’s amino acid sequence around the substitution site across mammalian species revealed that the p.Thr62Met substitution resided in a highly conserved region. The SIFT analysis indicated that the p.Thr62Met (SIFT score = 0.05) was most likely not tolerated. Thus, this amino acid change was considered highly deleterious to the structure and function of the F8 protein.

### Vector administration and immune responses to the vector capsid

Vector administration was well tolerated, with no adverse events noted during the 2 days of hospitalization. No clinical abnormality was observed during the 31 months and 24 months of follow-up for dogs 1 and 2, respectively, following vector injection. Liver enzymes (ALT, AST, ALP, and GGT) remained within reference range for both dogs following gene therapy, except for a mild transient increase in ALT (215 U/L; reference range 16–91) noted 18 months after vector administration in Dog 2, with the ALT decreasing to 58 U/L when rechecked at 22 months post-treatment. Dog 2 was not exhibiting any clinical signs at the time of the ALT increase. Following AAV delivery, both dogs developed a sustained and robust immune response to the vector capsid determined by the increased titers of neutralizing antibodies to AAV8 capsid (Fig 2), observed previously in preclinical and clinical studies using AAV vectors [10,12,14,24].

### Liver-gene therapy resulted in elevated levels of circulating cFVIII-BDD without antibody formation to the transgene

For Dog 1, circulating plateau levels of cFVIII-BDD are 1.6–2% of normal, without formation of antibodies to the transgene (Table 2, Fig 1). At 7 weeks following vector administration, the dog developed a hematoma on his neck after playing roughly with other dogs, however, the hematoma resolved quickly without administration of blood products. At 5 months following vector administration, the dog sustained a bite wound that resulted in a large hematoma necessitating transfusion support (1 unit packed red blood cells and 8 units of cryoprecipitate, as well as an infusion of cFVIII-BDD protein at dose of 50 IU/kg). One year later, a spontaneous bleeding episode (hematoma in the forelimb) occurred, and infusion of cFVIII-BDD protein at dose of 50 IU/kg was sufficient to control the bleeding. No adverse effects were noted upon the protein infusion at either time point. Approximately 5 months after the most recent protein injection, screening tests for inhibitory antibodies using Bethesda assay and non-inhibitory antibodies using anti-canine specific cFVIII-BDD were negative.

**Table 2:** Summary of liver gene therapy by AAV8 encoding canine FVIII in privately owned severe hemophilia A dogs.

Clinical Characteristics				Prior to AAV injection (41 months follow-up total)				Post AVV injection (55 months follow-up total)^b^			
Dog	Breed	Age^a^	Body weight (kg)	FVIII activity	Spontaneous bleeding episodes	Traumatic bleeding episodes	Transfusion events	FVIII activity	Spontaneous bleeding episodes	Traumatic bleeding episodes	Transfusion events
1	German shepherd mix	13	22	< 1%	11	1 (neuter)	10	1.6–2%	2	1 (bite wound)	2
2	Stafford-shire bull terrier	28	31	< 1%	11	2 (sutures, microchip)	16	1%	1	0	0

^a^Age in months at the time of vector administration.

^b^Follow-up period of 31 months for Dog 1 and 24 months for Dog 2.

For Dog 2, circulating levels of cFVIII-BDD remain stable at 1% of normal with no evidence of inhibitors to cFVIII (Fig 1). This dog received no additional injection of cFVIII protein or any other blood product following vector administration.

### Sustained improvement of the disease phenotype following AAV gene therapy

In both dogs a substantial reduction (90%) was observed in spontaneous bleeding episodes during follow-up period of 31 and 24 months for Dog 1 and Dog 2, respectively (Table 2). During the cumulative period of 55 months post-AAV administration, three spontaneous bleeding episodes (two in Dog 1 and one in Dog 2) were documented, whereas the medical records prior to gene therapy confirmed 22 episodes in 41 months in both dogs. These data are consistent with the number of visits to the hospital prior to and after AAV injection. The modest relative increase in FVIII activity following AAV gene therapy was sufficient to prevent most spontaneous bleeding in dogs with severe HA, consistent with a moderate phenotype of the disease.

## Discussion

Hemophilia is a relatively common disease among privately owned dogs, with an estimated 60 new cases diagnosed each year in the US through the Comparative Coagulation Laboratory at Cornell University (personal communication, Marjory Brooks, 2015). Although the prevalence of HA in dogs in unknown, most of these cases are HA, as in humans, suggesting that an endogenous mechanism of mutation in the *F8* gene is relatively common among mammals, as naturally occurring HA has also been reported in rats [25], sheep [26], cats, and horses (review in [27]).

Worldwide, more than 2,000 *F8* mutations have been identified in humans with HA, and the intron 22 inversion is the most common mutation in patients with severe disease. The advent of emerging novel technologies is likely to identify mutations in those patients (4%) without an as yet recognized molecular defect in the *F8* gene [28,29]. Interestingly, in an AAV liver gene therapy for hemophilia B, one human subject without a mutation in the *F9* coding region and no history of inhibitor was enrolled, and no adverse events were reported. However, the risk of formation of inhibitor to FIX is 5-fold lower than FVIII [1]. Similar to humans, the intron 22 inversion has been identified as the underlying mutation in the canine *F8* gene in two of three well characterized severe HA canine models in North America [30,31]. The use of outbred HA dogs with a distinct missense mutation or without an underlying mutation provides a unique opportunity to gain further insights into the risk of immune responses to FVIII in gene and protein therapy.

The challenge in providing effective care of these fragile, privately owned dogs with severe hemophilia, having an unpredictable onset of spontaneous bleeding and commonly trauma-induced hemorrhage, is substantial. The relatively short half-life of cFVIII-BDD (~ 8–12 hours), limited access to plasma products enriched in cFVIII (such as cryoprecipitate), and the lack of commercially available recombinant cFVIII protein also contribute to the difficulty in adopting replacement therapy in dogs, as for humans. Our successful experience with research colonies in achieving therapeutic levels of clotting factors (FIX or FVIII) following a single injection of AAV vectors for liver (n = 20) [11,14,16,24] or skeletal muscle (n = 40) [32] expression of the transgene motivated the extension of this strategy to pet owners with severe clinically affected dogs. Sustained expression of circulating FIX over a period of 3–9 years in dogs with severe HB prone to inhibitor development (n = 4) [14,24,33] suggests that liver gene therapy has the potential for phenotype improvement but without increased immunogenicity. The ability to eradicate pre-existing neutralizing antibodies to canine FVIII (n = 4) [14] or FIX (n = 1) [24] protein following AAV gene therapy highlights the potential for transgene-specific immune tolerance induction, further supporting the safety of this strategy for outbred dogs with naturally occurring hemophilia.

In this long-term follow up of two adult dogs with severe HA treated with gene transfer, the sustained expression of therapeutic levels of cFVIII-BDD (~1%) was associated with a substantial reduction in expected bleeding episodes (90%) and, therefore, an improvement of the disease phenotype, consistent with moderate hemophilia. One dog (Dog 1) required blood component therapy and an injection of cFVIII-BDD protein for management of severe bleeding associated with a bite wound and later an injection of cFVIII-BDD protein alone for the management of a spontaneous hematoma. Notably, no antibody formation to cFVIII was detected in these dogs, thus, these findings suggest that liver gene therapy is safe in outbred dogs with HA.

Because these dogs live in their natural home environment and, therefore, are more frequently exposed to trauma and other hemostatic challenges than animals in research colonies [13,14], the number of bleeding episodes for Dog 1 prior to gene therapy was almost double that expected for colony dogs (11–12 *vs*. 5–6 bleeds/year, respectively). However, the bleeding episodes in privately owned and research dogs with severe HA are reduced similarly after endogenous cFVIII-BDD expression by AAV. This improvement in the disease phenotype is also consistent with the data from hemophilic dogs from research colonies following gene therapy [13,32] and with the prophylactic replacement therapy that is the standard care of humans with hemophilia in the developed world. Interestingly, both dogs had low titers of neutralizing antibodies to AAV8 capsid (<1:5), currently an inclusion criteria for clinical trials using intravascular delivery of AAV vectors [12,22,23]. High neutralizing antibody titers (> 1:5) could prevent efficient gene therapy by intravascular delivery of AAV8. Thus, vector readministration of the same serotype is not feasible [10,12,22,23], although some limited success in gene expression has been observed in dogs by using alternative AAV serotypes [34]. Of note, approximately 30% of the general human population has detectable levels of neutralizing antibodies to AAV8 [35,36] and, thus, cannot be enrolled in gene therapy trials using a similar serotype. To date, attempts to eradicate these antibodies using plasmapheresis and/or immune suppression are often associated with poor outcomes and not currently used in clinical trials [37–39].

Collective data showed that AAV8 vectors transduced human hepatocytes >10 times less efficiently than murine hepatocytes. Similar data were also observed in non-human primates [40]. In terms of predicting the efficacy of a given strategy based on AAV liver gene therapy, the dog model is potentially the most promising. In our early studies on AAV2, preclinical studies in dogs were predictive of the therapeutic dose in humans [10,11]. Recently, in dogs AAV8 at doses of 1 x10^12^ vg/kg resulted in 3% of FIX antigen [24] which is closer to findings in humans (2 x 10^12^ vg/kg) of 5.1±1.7% of normal [12] than FIX antigen levels above 200% in non-human primates [41].

In summary, to our knowledge, this is the first report of gene therapy in privately owned dogs with HA resulting in a significant improvement of the disease phenotype after a single vector injection, resembling the success of early phase clinical trials for humans with HB. This report also highlights that the use of preclinical animal models of disease has not only a direct impact on the development of therapies for a variety of diseases, including hemophilia, affecting humans, but allows the improvement of the health of animals in their home environment.
